# Towards microbiome transplant as a therapy for periodontitis: an exploratory study of periodontitis microbial signature contrasted by oral health, caries and edentulism

**DOI:** 10.1186/s12903-015-0109-4

**Published:** 2015-10-14

**Authors:** Alex E. Pozhitkov, Brian G. Leroux, Timothy W. Randolph, Thomas Beikler, Thomas F. Flemmig, Peter A. Noble

**Affiliations:** Department of Oral Health Sciences, University of Washington, Box 3574444, Seattle, WA 98195-7444 USA; PhD Program in Microbiology, Alabama State University, Montgomery, AL 36101 USA; Fred Hutchinson Cancer Research Center, 1100 Fairview Ave. N., PO Box 19024, Seattle, WA 98109 USA; Section of Periodontics, School of Medicine, Heinrich-Heine-University, Moorenstrasse 5, 40225 Düsseldorf, Germany; Faculty of Dentistry, The University of Hong Kong, Prince Philip Dental Hospital, 34 Hospital Road, Sai Ying Pun, Hong Kong, SAR Peoples’ Republic of China

**Keywords:** Bacteriotherapy, Microbial transplant, Caries, Edentulism, Periodontitis, Red complex

## Abstract

**Background:**

Conventional periodontal therapy aims at controlling supra- and subgingival biofilms. Although periodontal therapy was shown to improve periodontal health, it does not completely arrest the disease. Almost all subjects compliant with periodontal maintenance continue to experience progressive clinical attachment loss and a fraction of them loses teeth. An oral *microbial transplant* may be a new alternative for treating periodontitis (inspired by fecal transplant). First, it must be established that microbiomes of oral health and periodontitis are distinct. In that case, the health-associated microbiome could be introduced into the oral cavity of periodontitis patients. This relates to the goals of our study: (i) to assess if microbial communities of the entire oral cavity of subjects with periodontitis were different from or oral health contrasted by microbiotas of caries and edentulism patients; (ii) to test *in vitro* if safe concentration of sodium hypochlorite could be used for initial eradication of the original oral microbiota followed by a safe neutralization of the hypochlorite prior transplantation.

**Methods:**

Sixteen systemically healthy white adults with clinical signs of one of the following oral conditions were enrolled: periodontitis, established caries, edentulism, and oral health. Oral biofilm samples were collected from sub- and supra-gingival sites, and oral mucosae. DNA was extracted and 16S rRNA genes were amplified. Amplicons from the same patient were pooled, sequenced and quantified. Volunteer’s oral plaque was treated with saline, 16 mM NaOCl and NaOCl neutralized by ascorbate buffer followed by plating on blood agar.

**Results:**

Ordination plots of rRNA gene abundances revealed distinct groupings for the oral microbiomes of subjects with periodontitis, edentulism, or oral health. The oral microbiome in subjects with periodontitis showed the greatest diversity harboring 29 bacterial species at significantly higher abundance compared to subjects with the other assessed conditions. Healthy subjects had significantly higher abundance in 10 microbial species compared to the other conditions. NaOCl showed strong antimicrobial properties; nontoxic ascorbate was capable of neutralizing the hypochlorite.

**Conclusions:**

Distinct oral microbial signatures were found in subjects with periodontitis, edentulism, or oral health. This finding opens up a potential for a new therapy, whereby a health-related entire oral microbial community would be transplanted to the diseased patient.

**Electronic supplementary material:**

The online version of this article (doi:10.1186/s12903-015-0109-4) contains supplementary material, which is available to authorized users.

## Background

The human oral microbiome is composed of a wide variety of microorganisms that play important roles in health and disease. Bacteria, for example, maintain oral and systemic health [1], but can also cause disease. Bacteriophages shape microbial diversity [2]. Protozoans and fungi consume food debris and other microbes [3, 4] and archaea utilize fermentation byproducts to produce methane [5]. Yet, in terms of microbial species, our current understanding of the human oral microbiome is limited to a few well-studied microorganisms. Based on what is known about these microbes, researchers have developed conceptual models with the explicit purpose of determining the etiology of oral diseases (e.g., [6, 7]). These models suggest putative interactions among the microorganisms as well as between microorganisms and the host. Although these studies have significantly advanced the field, recent studies show that the oral cavity is a complex and dynamic habitat consisting of hundreds of different interacting species [8, 9].

The conceptual model for periodontitis is that *Porphyromonas gingivalis, Tannerella forsythia, Treponema denticola* and *Aggregatibacter actinomycetemcomitans* are responsible for the disease [6, 10, 11]. Evidence from animal models indicated that these bacteria may disrupt tissue homeostasis by manipulating signaling pathways of the host and once the innate immunity is compromised, cause a shift in the relative abundance of microbes, resulting in inflammation and bone loss [12, 13]. Yet, it is well established that these bacteria i.e., *P. gingivalis, T. forsythia, T. denticola, A. actinomycetemcomitans*, are also found in healthy oral cavities (e.g., [14–19]). Moreover, *P. gingivalis* is not found in up to half of the patients with chronic or aggressive periodontitis [20]. The paradox of these microbes being present in both health and disease questions the validity of the conceptual underpinnings of the etiology of periodontitis, which invites for alternative ways of thinking about periodontitis as well as other oral diseases.

Conventional periodontal therapies aim at controlling supra- and subgingival biofilms and managing prognostic factors such as poor glycemic control in subjects with diabetes mellitus and active smoking [21, 22]. Although periodontal therapy has been shown to improve periodontal health and reduce the rate of further clinical attachment loss [23, 24] and tooth loss due to periodontitis [25–27], it falls short of completely arresting the disease. Almost all subjects compliant with periodontal maintenance care continue to show signs of progressive clinical attachment loss, and one- to two- thirds of them, lose one or more teeth during an extended period of periodontal maintenance care [28, 29]. We believe new thinking is necessary for finding successful ways of treating periodontitis.

The knowledge from a different field indicates that an appropriate microbial community is a key for maintaining resistance against infection. A classic example is the gut microbiome, which protects the host from *Clostridium difficile* (CD) infection. Specifically, some patients develop CD colonization of their gut upon antibiotic treatment, which eradicates out their innate microbiome. A successful strategy for combating CD by transplanting gut microbiota from a healthy donor was first medically recorded more than 50 years ago [30]. (The origins of the procedure date back to the 4^th^ century in China [31]). Specifically, a suspension of stool taken from a systemically healthy intimate partner, relative or friend is introduced into the gastrointestinal tract of the recipient via colonoscopy, enema or a nasogastric tube. Recent systematic review reports approx 90 % efficiency of eradicating the CD infection by a fecal transplant [32]. Besides the CD infection, other potentially dysbiotic diseases were found to be responsive to the microbial transplantation, such as inflammatory bowel disease, obesity. For a review on current developments in the gut microbial transplant see Kelly et al. [33].

Inspired by the success of the fecal transplant, here we put forward a concept of a microbial transplant as a potential therapy for periodontitis. We envision the therapy of consisting of three steps: (i) harvesting sub- and supra-gingival microbiota from a healthy donor, e.g., spouse or a partner; (ii), performing deep cleaning, root planning and applying a broad-spectrum antimicrobial agent to the periodontitis patient; and (iii) neutralizing the antimicrobial agent immediately following by a rinsing with a microbial suspension harvested from the healthy donor in the periodontitis patient.

The objectives of the present study were two-fold. The first objective was to assess if the entire oral microbial community of periodontitis was different from established caries, edentulism, and oral health. The second objective was to test an approach for applying a broad-spectrum safe antimicrobial agent (NaOCl) to significantly reduce the load of existing microbiome followed by neutralization of this agent for subsequent microbial transplantation.

## Methods

### Study subjects

Adult subjects were recruited at the Department of Periodontics, University of Washington, Seattle, WA, U.S.A. and Section of Periodontics, University of Düsseldorf, Germany. A written informed consent for participation in the study was obtained from the subjects. The study was approved by the University of Washington Institutional Review Board, ref. number 3570. Subjects were enrolled if they had one of the following clinical conditions: severe periodontitis, caries, edentulism, or oral health. A periodontitis case was defined as having at least 2 interproximal sites at different teeth with clinical attachment loss (CAL) of 6 mm or greater and at least 1 interproximal site with probing depth (PD) of 5 mm or greater [34] and a minimum of 20 permanent teeth, not including 3^rd^ molars. Subjects were excluded from the periodontitis group if they had multiple established caries lesion or wore a removable partial denture. A caries case was defined as having the following number of teeth with established caries lesions: 6 or more teeth in subjects 20 to 34 years of age; 4 or more teeth in subjects 35 to 49 years of age; and 3 or more teeth in subjects 50 years of age and older. Established caries was defined as a class 4 lesion according to the International Caries Detection and Assessment System. The number of teeth with caries lesion in caries cases was greater than one standard deviation above the mean of caries extent in respective age group the U.S.A. [35]. Exclusion criteria for a caries case were interproximal sites with CAL of 4 mm or greater or PD of 5 mm or greater [34]. An edentulous case had to be completely edentulous in both jaws and their teeth had to be extracted more than one year before the enrollment in the study. A healthy case was defined as having 28 teeth, not counting 3^rd^ molars, or 24 or more teeth, not counting 3^rd^ molars if premolars had been extracted for orthodontic reasons or were congenitally missing with no signs of oral disease. Exclusion criteria for a healthy case included: smoking, loss of permanent teeth due to caries or periodontitis, any interproximal sites with CAL of 4 or greater or PD of 5 mm or greater, or any established caries lesions. Exclusioncriteria for all groups included: oral mucosal lesions, systemic diseases, and use of antibiotics or local antiseptics within 3 months prior to the study.

### Sample collection

For all but the edentulous patients, supra- and subgingival plaque was collected from six sites with the deepest probing depth in each sextant. One sterile paper point per site was inserted into the deepest aspect of the periodontal pocket or gingival sulcus. Biofilm from oral mucosae was collected by swiping a sterile cotton swab over the epithelial surfaces of the lip, left and right buccal mucosae, palate, and dorsum of the tongue [36]. Samples were stored at −80 °C.

### Molecular methods

Microbial DNA was isolated from cells by physical and chemical disruption using zirconia/silica beads and phenol-chloroform extraction in a FastPrep-24 bead beater [37]. Prokaryotic 16S rRNA genes were amplified using universal primers (27 F and 1392R) using the GemTaq kit from MGQuest (Cat# EP012). The PCR program involved a pre-amplification step of 10 cycles with annealing temperature of 56 °C followed by 20 amplification cycles with annealing temperature 58 °C. In each cycle, elongation time was 1 min 10s, at 72 °C. PCR was finalized by extended elongation for 5 min. PCR products were purified with DNA Clean & Concentrator columns (Zymo Research, USA) and quantified using the NanoDrop (Agilent, USA).

Due to technical reasons, for some subjects, subgingival and mucosal microbiotas were pooled together into one vial, while for other subjects the subgingival and mucosal microbiotas were stored separately. Since our goal was to investigate the entire microbial community, for the separately stored supra and subgingival microbiota samples, equal quantities of PCR product derived from swab and paper point samples were pooled together for each patient. For edentulous patients, there were no paper point samples. Each purified PCR product, 500 ng, was labeled with a Multiplex Identifier (MID) during the Roche Rapid Library preparation step. Four MID-tagged sequences, representing each of the conditions, were combined in equimolar concentrations and subjected to emPCR and DNA sequencing protocols as specified by the manufacturer’s recommendations for the Roche 454 Jr. instrument.

### Data analysis

The obtained sequences were separated out by their respective Multiplex Identifier (MID) and uploaded to the MG-RAST web server [38]. The MG-RAST pipeline assessed the quality of sequences, removed short sequences (multiplication of standard deviation of length cutoff of 2.0) and removed sequences with ambiguous bp (non-ACGT; maximum allowed number of ambiguous base pair was set to 5). The pipeline annotated the sequences and allowed the integration of the data with previous metagenomic and genomic samples. The RDP database was used as annotation source, with minimum sequence identity of 97 %, maximum e-value cutoff at 10^−5^, and minimum sequence length of 100 bases. Alpha diversity analysis was conducted in MG-RAST.

Orthogonal transformation of the annotated rRNA genes to their principal components (PC) was conducted using normalized abundances [39, 40]. Normalization of the abundance was performed identically to the procedure used by MG-RAST. Specifically, abundances were increased by one, log2 transformed, and centered to produce relative values. Relative values were standardized by dividing them by the standard deviation of the log2 values [38]. The data were graphed on a 2 dimensional ordination plot. To determine the relative contribution of the microbial species to the plot, we let X denote the 16 by 578 (patients by species) matrix of the normalized abundance values. The matrix X was used to produce a 16 × 16 matrix D of distances between all pairs of subjects. Principal coordinate analysis (PCoA, i.e., multidimensional scaling) was achieved by performing principal component analysis (PCA) on the matrix of distances, D. The Euclidean distance metric was used to create this matrix, but this metric produced results similar to those when the alternative Bray-Curtis distance was used. To investigate and visualize differences between the four patient groups, the first two principal components, PC1 and PC2, of the distance matrix D were retained. To establish which species were most prominently responsible for the groupings, the projection of each species onto the (PC1, PC2) plane was calculated; those species with the largest projections are displayed in the right panel of Fig. 3. That is, this figure shows a biplot of the species most highly correlated PC1 and PC2.

Mann–Whitney test (alpha = 0.05) was used to investigate significant difference in normalized relative abundances. The abundance data were normalized in two different ways: MG-RAST normalization as described above and raw abundances normalized to the total number of reads in a sample. Differences in alpha diversities by condition were determined using ANOVA. Two-tailed T-tests, assuming unequal variance, were used investigate if there was significantly different in the means (alpha = 0.05). These analyses were performed using SAS JMP.

### Sodium hypochlorite experiments

According to previously suggested concentration [41], sodium hypochlorite experiments were conducted with a 1:50 dilution of the 6 % household bleach (Chlorox, The Clorox Company, USA), the “hypochlorite working solution”. The dilution corresponds to 16 mM NaOCl. The neutralizing agent was a sodium ascorbate buffer produced by adjusting pH of a 23 mM ascorbic acid solution with concentrated NaOH until pH 5.3.

Three samples of dental plaque from a volunteer was subjected to three challenges: (i) resuspension in saline, (ii) resuspension in the hypochlorite working solution and (iii) resuspension in the hypochlorite working solution that was previously neutralized with equal volume of the ascorbate buffer. Resuspended plaque samples were plated on blood agar plates.

Active chlorine concentration was crudely assessed by iodine colorimetry. A colorimetric solution contained 1 volume of 0.1 % stabilized starch solution (Fisher Scientific) mixed with 0.1 volume of 1 M potassium iodide. Two volumes of the colorimetric solution were mixed with 1 volume of the solution containing NaOCl. Intense blue color indicated detectable active chlorine. Iodine colorimetry was calibrated with a dilution series of the hypochlorite working solution with the following dilutions 1:200, 1:400, 1:800, 1:1600, 0. The dilution1:800 still showed a hint of blue, while 1:1600 was completely colorless.

## Results

### Demographics

A total of 16 subjects, 4 subjects with each of the assessed oral conditions, were enrolled into the study. Ten subjects were from Germany and 6 from the U.S.A.; 9 were females and 7 males. The age of the enrolled subjects ranged from 28 to 92 years. Subjects with periodontitis had a median of 34 sites (range 15 to 48) with PD of 4 to 6 mm and 5 sites (range 4 to 26) with PD of 7 mm or greater while none of the subjects with caries and none of the healthy subjects had any sites with PD of 4 mm or greater. Subjects with caries had a median of 14 teeth (range 9 to 17) with established caries while periodontitis and healthy subjects were mostly caries free (Table 1).

**Table 1:** Demographics of the patients population

	Condition			
Parameter	Healthy	Periodontitis	Caries	Edentulous
America/Europe	0/4	2/2	2/2	2/2
Male/Female	2/2	2/2	2/2	1/3
Ages	41 (31–52)^a^	47 (28–58)	39 (29–49)	77 (58–92)
Number of teeth	27 (25–28)	28 (22–30)	28 (22–31)	0
ICDAS ≥ 4	0	0 (0–1)	14 (9–17)	--
PD ≤ 3 mm	100	62 (26–79)	100	--
PD 4–6 mm	0	34 (15–48)	0	--
PD ≥ 7 mm	0	5 (4–26)	0	--

*ICDAS* International Caries Detection and Assessment System, *4* denotes established decay (dentine shadow), *PD* pocket depth

^a^Median (min-max)

### Microbial signatures

**Fig. 1:**
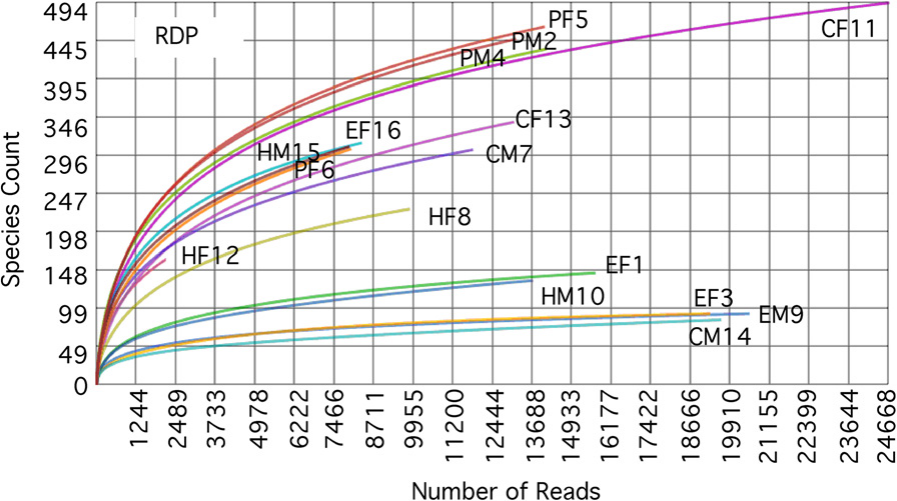
Rarefaction curves obtained using RDP database with 97 % similarity, 100 bp minimum alignment and e-value of 10^−5^. See Table 2 for labels

The average (± std) number of 16S rRNA amplicons sequences per individual oral microbiota was 13,104 ± 5,533 (Additional file 1: Table S1). The length and GC content of the sequences was similar for all individuals: 514 ± 10 bp and 53 ± 1 bp, respectively. Comparison of the number of sequences, sequence length, or GC content revealed no significant differences by oral condition or gender, indicating a balanced data set. The rarefaction curve of most samples approached saturation, indicating sufficient reads for comparisons by condition and gender (Fig. 1).

**Fig. 2:**
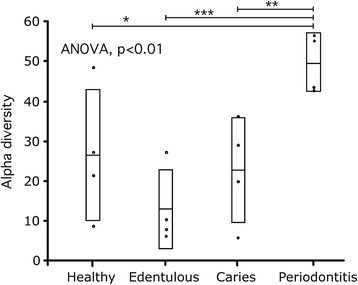
Microbial species diversity differences among four oral conditions. Shown are raw data, mean and standard deviation. (*p = 0.06, **p < 0.05, ***p < 0.001, *T*-test)

**Fig. 3:**
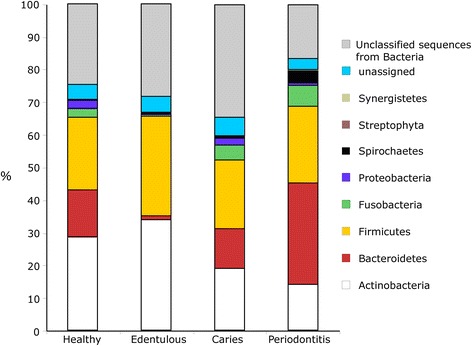
Percent abundance of microbes by phylum and condition

Alpha diversity of the oral microbiome in periodontitis subjects was significantly (p < 0.05) greater compared to that found in subjects with caries and edentulous subjects. Although the oral microbiome was more diverse in periodontitis subjects compared to healthy subjects, the difference missed statistical significance (p = 0.06) (Fig. 2). The composition of the oral microbiome in subjects with periodontitis was distinctly different from that of all other oral conditions. Specifically, the periodontitis condition had greater abundances of Bacteroidetes (32 %), Fusobacteria (7 %), Spirochaetes (5 %) and Synergisetes (1 %) and fewer Actinobacteria (14 %) (Fig. 3). Actinobacteria were most abundant in the oral microbiome of healthy (28 %) and edentulous subjects (34 %), followed by Firmicutes, which occurred in abundances of 22 % and 30 %, respectively (Fig. 3). Subjects with caries showed somewhat lower abundances of Actinobacteria (19 % vs. 29 %) and slightly greater abundances of Fusobacteria (5 % vs. 3 %) in the oral microbial communities compared to healthy subjects (Fig. 3).

#### “The red complex”

**Fig. 4:**
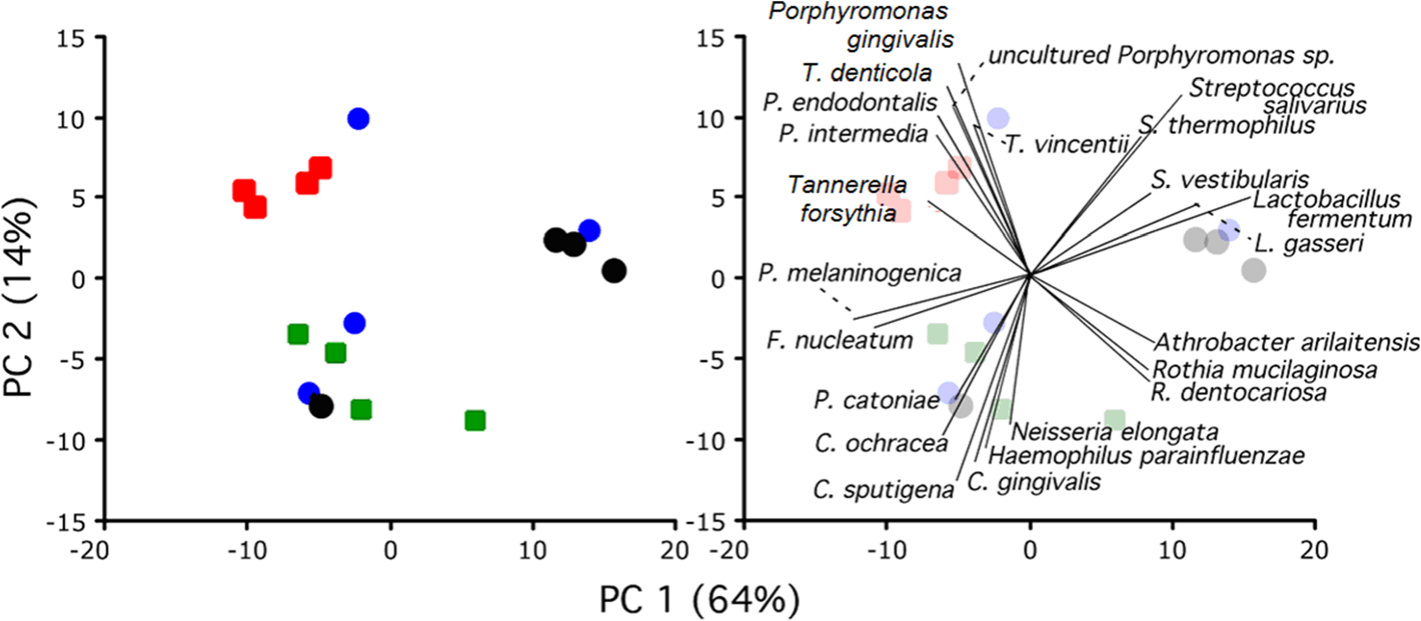
*Left* panel: PCoA ordination plot of the 16 human oral samples by condition. Condition: *red*, periodontitis; *blue*, caries; *black*, edentulous; *green*, healthy. *Right* panel: Projection of major microbial species contributing to the groups

An ordination plot based on the 16S rRNA genes revealed that the oral microbiomes in subjects with periodontitis, edentulism, or oral health formed distinct groups (Fig. 4, left panel). The ordination plot explained 78 % of the observed variability. These results, along with the high diversity values, suggest that oral microbiome in subjects with periodontitis, edentulous subjects, and healthy subjects were very different from each other. Permutation MANOVA (pseudo F test, refs. [42, 43]) showed significant differences in microbial species abundances between the four oral conditions (Bonferroni-adjusted p-value of 0.0083). The Bonferroni adjustment refers to the 6 comparisons, i.e., all pairs of the 4 groups. In order to determine if the “red complex” bacteria (*P. gingivalis*, *T. denticola* and *T. forsythia*) were absolute determinants for the microbial signature of periodontitis, the abundances of these organisms were removed from the original data set. Non-parametric MANOVA and ordination plot analyses of the data set without “red complex” bacteria did not alter the findings shown in Fig. 4a or the p-value.

Projecting the top 23 contributing bacteria on the PCoA plot (i.e., biplot) shows the relative contribution of bacteria to the ordination (Fig. 4, right). Since the purpose of the biplot was predominantly illustrative, the number of displayed species, 23, was chosen arbitrary based on the upper tail of the histogram of projection sizes (Additional file 1: Figure S1). *P. endodontalis, Prevotella intermedia, T. vincentii,* and an uncultured *Porphyromonas* sp. were found to contribute to the microbial composition in periodontitis while a complex of *P. melaninogenica, Fusobacterium nucleatum, P. catoniae, Capnocytophaga ochracea* and *C. sputigena, C. gingivalis, Haemophilus parainfluenzae and Neisseria elongata* contributed to the microbial composition of orally healthy subjects. Members of the *Streptococcus* and *Lactobacillus* were associated with edentulism.

The species contributing to the differences in diversity and the groupings in the ordination plot are shown in Table 2. Twenty-nine out of the 587 microbial species had higher abundances in patients with periodontitis than those in the other assessed conditions. In the healthy subjects, 10 out of the 587 microbial species were found in higher abundances compared to the non-healthy subjects (Table 3).

**Table 2:** Bacterial species that had significantly different abundances (%) between individuals with the non-periodontitis (NP; e.g., healthy, caries, edentulous) and periodontitis (P) abundances based on Mann–Whitney test (alpha = 0.05)

Phylum/class	Genus/species	NP	P
Actinobacteria	*Atopobium vaginae*	0.01	0.35*
	*Actinomyces georgiae*	0.05	0.35**
	*Actinomyces meyeri*	0.10	0.49**
Bacteroidetes	*Porphyromonas endodontalis*	0.12	2.91*
	*uncultured Porphyromonas sp.*	0.04	1.26*
	*Porphyromonas gingivicanis*	0.00	0.04**
	*Tannerella forsythia*	0.20	0.83*
	*Prevotella intermedia*	0.12	3.23*
	*Prevotella pallens*	0.14	0.85*
	*Prevotella pleuritidis*	0.00	0.01**
	*Prevotella salivae*	0.20	0.74**
Firmicutes/Clostridia	*Parvimonas micra*	0.12	1.19*
	*Eubacterium saburreum*	0.07	0.28*
	*Pseudobutyrivibrio xylanivorans*	0.00	0.06*
	*Peptostreptococcus anaerobius*	0.07	0.50*
Firmicutes/Negativicutes	*Megasphaera micronuciformis*	0.12	0.92*
Fusobacteria	*Leptotrichia wadei*	0.10	0.32**
Proteobacteria/Deltaproteobacteria	*uncultured Desulfobulbus sp.*	0.00	0.02*
Proteobacteria/Epsilonproteobacteria	*Campylobacter rectus*	0.03	0.14*
	*Campylobacter concisus*	0.03	0.07**
Spirochaetes/Spirochaetales	*Treponema denticola*	0.07	1.60*
	*Treponema maltophilum*	0.01	0.25*
	*Treponema medium*	0.03	0.29*
	*Treponema socranskii*	0.05	0.21*
	*Treponema sp.*	0.03	0.10*
	*Treponema vincentii*	0.07	0.71*
	*Treponema pectinovorum*	0.00	0.04**
Synergistetes	*Synergistetes bacterium SGP1*	0.05	0.23*
	*Aminobacterium colombiense*	0.01	0.05*

*Significant difference using both normalization methods (i.e., raw species abundances were log2 transformed, normalized to produce relative values ((raw values-average)/std) versus abundances normalized to the total number of reads in a sample)

**Significant difference for abundances using data normalized only to the total number of reads in a sample

**Table 3:** Microbial species that had significantly different abundances (%) between individuals with the non-healthy (NH; periodontitis, caries, edentulous) and healthy (H) abundances based on Mann–Whitney test (alpha = 0.05)

Phylum/class	Genus/species	NH	H
Actinobacteria	*Micrococcus lylae*	0.00	0.03**
Bacteroidetes	*Prevotella marshii*	0.01	0.09**
Firmicutes/Bacilli	*Abiotrophia para-adiacens*	0.22	0.60**
	*Granulicatella elegans*	0.20	0.66*
	*Granulicatella adiacens*	0.60	1.27**
	*Streptococcus iniae*	0.04	0.15*
Firmicutes/Negativicutes	*Selenomonas ruminantium*	0.00	0.10*
Proteobacteria/Betaproteobacteria	*Neisseria polysaccharea*	0.00	0.02*
Proteobacteria/Gammaproteobacteria	*Aggregatibacter segnis*	0.01	0.21**
	*Haemophilus parainfluenzae*	0.17	0.46*

*Significant difference determined using both normalization methods (i.e., raw species abundances were log2 transformed, normalized to produce relative values ((raw values-average)/std) versus abundances normalized to the total number of reads in a sample)

**Significant difference for abundances using data that was normalized to only the total number of reads in a sample

### Antimicrobial agent and its neutralization

**Fig. 5:**
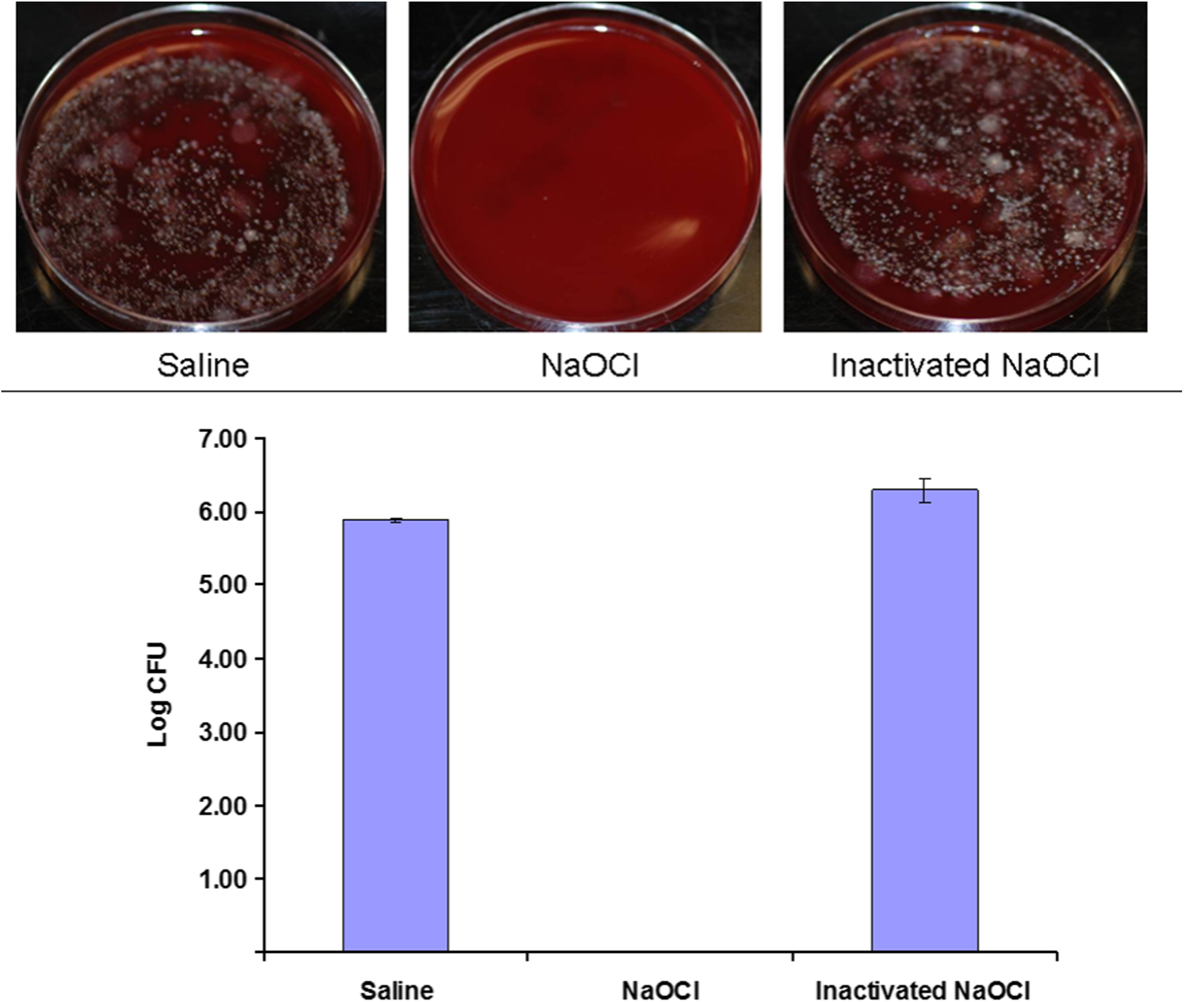
Dental plaque treated with saline, the hypochlorite working solution or inactivated hypochlorite working solution

Antimicrobial activity of sodium hypochlorite was tested with a volunteer’s dental plaque. Resuspension of the plaque in 1:50 dilution of the household bleach, i.e., “the hypochlorite working solution” resulted in a complete inhibition of bacterial growth on a blood agar. In contrast, the viability of whole dental plaque bacteria was maintained following exposure to the hypochlorite working solution inactivated for 7 min with 23 mM sodium ascorbate buffer (Fig. 5). Starch-iodine colorimetry assay revealed that sodium ascorbate - ascorbic acid buffer reduced the concentration of active chlorine by a factor of at least 800–1600, i.e., below 10–20 uM, after 30 s of inactivation.

## Discussion

### Potential for the periodontitis bacteriotherapy

The first step towards developing a bacteriotherapy for periodontitis is to establish the existence of a microbial community characteristic of periodontitis as well as the community characteristic of oral health. Two additional communities from edentulous and caries patients, whose microbial communities are expected to be distinct, were added for contrasting purposes.

Several studies have compared microbial communities isolated from different sites in the oral cavity and from patients with different clinical conditions (e.g., [14, 15, 17, 19, 44–47]). One of these studies revealed distinct partitioning of bacterial communities in subgingival biofilms from healthy and diseased periodontal sites [45]. Another study indicated that not only the oral microbial species were drastically different between periodontal health and disease but also their gene expression [48]. Moreover, in subjects with periodontitis, disease-associated bacteria were found on the buccal mucosae, tongue, and saliva indicating that the entire oral microbiome might be altered [49–53].

Since the bacteriotherapy would likely be performed by attempting to transplant both sub- and supra-gingival microbes, the present study was set to test the hypothesis that overall compositions of the oral microbial communities were distinct between the conditions of periodontitis, edentulism, caries, and oral health. In fact, the entire oral microbiome of each subject was obtained by pooling together several oral cavity sub-microbiomes such as subgingival, supragingival, and mucosal membranes.

The key finding of this study, critically important for the potential bacteriotherapy, is that microbial compositions (i.e., signatures) of the entire (or substantial subset of) oral microbiomes of subjects with periodontitis contrasted by edentulism and oral health are distinct. In addition, the microorganisms which are usually considered as pathogens of periodontitis, *P. gingivalis*, *T. denticola* and *T. forsythia*, are not the sole representatives of the periodontitis microbial signature. In particular, this study provides evidence that a broad microbial-community model of oral health may offer a valuable perspective when considered in conjunction with (or in contrast to) more common single-pathogen models ([7] and refs. therein). With this perspective, it is reasonable to expect that microbial community composition is a function of interspecies competition and cooperation, which provides optimism for further developing the bacteriotherapy. Below, we expand on the motivation for choosing molecular methods and sampling procedures.

### Methodological validity

Historically, subgingival oral microbiome was predominantly assessed by a checkerboard hybridization technique revealing signals from approx. 40 species (for review, see [54]). Pathogenicity of a few species, i.e., “colored complexes”, was presumed to account for oral conditions, such as periodontitis. With the advent of next generation sequencing, extensive cataloging of the subgingival microbiome has been conducted (e.g., [17, 45, 55]). In our study we also utilized high-throughput sequencing technology, because it provides an in-depth qualitative and quantitative characterization of the microbial communities, which is essential for discovery of microbial signatures.

The data for the analysis of microbial signatures were the relative abundances of microorganisms measured by the number of sequencing reads obtained from high-throughput sequencing of each subject’s oral microbiome. Contrary to what one might expect, it is important to note, that absolute abundances are not attainable by the high throughput sequencing due to the biases in amplification and other physicochemical reasons [56, 57]. After sequencing reads are processed, each subject is represented by a p-dimensional vector (p = 597) in the multidimensional space, with each coordinate corresponding to the relative abundance of each microbial species. The ordination analysis [58] presented in the Results section is a standard dimension-reducing method, which reveals the structure of the 3 groups (Fig. 4). Interpretation of these groups is based on the subjects’ microbial abundance profiles that are most similar and hence grouped closer to each other. Although the sample size is rather small (n = 16), the quality of the clusters and their statistical significance (based on permutation) suggests that the observed grouping may be reproducible in a larger, independent cohort. Other researchers, e.g., Kumar et al. [47], showed with a larger group that there is a clear separation between microbial communities for healthy and periodontitis and peri-implantitis patients. Another potential issue with generalization of our findings could be due to the fact that all health subjects were from Europe. Nevertheless, as was discovered by Nasidze et al. [59], the compositions of the oral microbial communities obtained from 12 worldwide locations of 10 individuals each were “larger among individuals from the same location than among individuals from different locations”. Hence the differences in the composition among individuals were not determined by geography.

### Periodontitis pathogenesis: “red complex” or entire community shift?

This study revealed grouping of subjects based on the composition of their entire oral microbiomes, which is consistent with previously reported results based on the microbial composition of their subgingival plaque samples [47]. Specifically, it has been suggested that there exist distinct microbial signatures characteristic of periodontitis, peri-implantitis, and oral health. Our research expands on these findings by investigating the entire oral microbiome including combined together subgingival, supragingival, and mucosal sub-microbiomes. Building upon a recent report by Griffen et al. [45], which showed a high microbial diversity of sub-gingival biofilms in subjects with periodontitis compared to oral health, our study also revealed a similarly increased diversity in the entire oral microbiome of subjects with periodontitis compared to edentulism, oral health and caries. Among the four conditions, our study showed the lowest microbial diversity in oral microbiomes of subjects with edentulism. This is presumably due to the absence of teeth and subgingival pockets in which otherwise mature biofilms develop [45]. It is important to note that there are other studies focused on microbial signatures of periodontitis (e.g., [60]), however these studies did not attempt to contrast several conditions.

Our observations support the polymicrobial nature of periodontitis contrary to the “old-school” long-standing notion of specific pathogens believed to cause periodontitis. Indeed, the oral microbial signature in periodontitis subjects included *P. gingivalis*, *T. denticola* and *T. forsythia*, which are collectively referred to as the “red complex”. As our results showed, red complex bacteria were not the absolute determinants of the periodontitis microbial signature. The presence of the red complex bacteria in oral health and other conditions could be attributed to differences in: (i) the host immune response, (ii) genes encoding virulence factors among different strains, and (iii) gene regulation [51]. The findings of distinct oral microbial signatures between subjects with periodontitis and oral health supported the notion that periodontitis is associated with a dysbiosis (i.e., microbial imbalance) of the entire oral microbiome. The oral dysbiosis may be due to the assembly of a synergistic microbial community that tilts the balance away from a health-associated microbial homeostasis [51]. It could be initiated by the acquisition of virulence factors by microbes and/or changes in the regulation/expression of genes within the community [17, 61]. Perhaps one or several microorganisms, e.g., *P. gingivalis*, are capable of initiating synergetic interactions that ultimately result in the emergence of disease-provoking microbial community [7], however a mechanistic proof is lacking.

The idea for the potential bacteriotherapy of periodontitis is inspired by the success of the fecal microbial transplant treatment of *Clostridium difficile* (CD) infection. It should be noted that while the diversity of bacteria associated with periodontal disease is very high [55, 62], the diversity of bacteria associated with CD infections is very low [63]. The reason to emphasize these differences is that the goal of bacteriotherapy for CD is to increase bacterial diversity whereas the goal of bacteriotherapy for periodontitis is not the same since the bacterial diversity is already high. Presumably in both diseases, replacement of healthy stable microbial communities is the answer to prevent future dysbiosis. However, without performing actual experiments, one cannot speculate on the success or failure of bacteriotherapy for periodontal disease.

### Antimicrobial treatment before the transplant

It is reasonable to assume that for the bacteriotherapy to be successful, the recipient’s oral cavity has to have lowest possible load of microorganisms to allow the new microbiome to flourish without interference from the previous disease-associated microbiome. Administration of adjunctive systemic antibiotics [64, 65] or a full-mouth disinfection approach that reduces the bacterial reservoir in all habitats of the oral cavity will result in more pronounced shifts in the microbial composition and significantly reduces the incidence of clinical attachment loss compared to supra- and subgingival debridement alone [37, 66, 67]. Still, it is desirable to administer such an antimicrobial agent that is effective against a broad spectrum of microbes and is easy to neutralize with another nontoxic agent, because as most antiseptics remain active in the oral cavity for quite some time [68, 69] and therefore, may affect the viability of an oral microbial transplant. We have been able to demonstrate that sodium hypochlorite (NaOCl), which is bactericidal against a panel of oral biofilm microorganisms [68, 70, 71], can be inactivated by a nontoxic sodium ascorbate – ascorbic acid buffer. Although some critics have said that NaOCl treatment is antiquated and dangerous, a recent study proved the opposite [72]. Moreover, the Galvan et al. study [72] showed safety and a significant reduction of plaque and bleeding on probing in the group that used NaOCl rinses compared to the control group.

## Conclusions

The present exploratory study revealed statistically significant microbial signatures of oral health and periodontitis. This finding presents a path forward for a potential new therapy for periodontitis, which could be based on substituting periodontitis-associated microbiome with the health-associated one. At the beginning (in 1950s), the fecal transplant therapy was successfully used without a deep mechanistic understanding. Currently, insights into the gene expression and metabolic effects of the therapy have been made (e.g., [73]). If the proposed therapy is proven to be successful, deep mechanistic studies will be of great interest.

## Additional file

Additional file 1:Table S1. Number of sequences obtained from each condition and sample. **Table S2.** Comparison of oral microbial community composition in different studies. **Figure S1.** Determination of an arbitrary cut-off for displaying species on a biplot (Fig. 4, right panel). (PDF 136 kb)

## Abbreviations

PCA: Principal component analysis
PCR: Polymerase chain reaction
MID: Multiplex identifier
PCoA: Principal coordinate analysis
bp: Base pairs
