# Supplementary material for: Towards microbiome transplant as a therapy for periodontitis: an exploratory study of periodontitis microbial signature contrasted by oral health, caries and edentulism
Source: BMC Oral Health. 2015 Oct 14;15:125. doi: 10.1186/s12903-015-0109-4 (PMC4607249; doi:10.1186/s12903-015-0109-4)
Supplement: Additional file 1:Table S1. — Number of sequences obtained from each condition and sample. Table S2. Comparison of oral microbial community composition in different studies. Figure S1. Determination of an arbitrary cut-off for displaying species on a biplot (Fig. 4, right panel). (PDF 136 kb) [file 12903_2015_109_MOESM1_ESM.pdf]

# SUPPLEMENTARY MATERIAL

# Towards Microbiome Transplant as a Therapy for Periodontitis: an Exploratory Study of Periodontitis Microbial Signature Contrasted by Oral Health, Caries and Edentulism.

Alex E. Pozhitkov[1*], Brian G. Leroux[1], Timothy W. Randolph[3], Thomas Beikler[4], Thomas F. Flemmig[5], Peter A. Noble[1,2]

*Corresponding author, Alex E. Pozhitkov, Email: pozhit@uw.edu

Authors' affiliations:

1. Department of Oral Health Sciences, University of Washington, Box 3574444, Seattle, Washington 98195-7444 Phone: 206-543-3641, Fax: 206-616-7478
2. PhD Program in Microbiology, Alabama State University, Montgomery, AL 36101
3. Fred Hutchinson Cancer Research Center, 1100 Fairview Ave. N. PO Box 19024 Seattle, WA 98109
4. Section of Periodontics, School of Medicine, Heinrich-Heine-University, Moorenstrasse 5, 40225 Düsseldorf, Germany.
5. Faculty of Dentistry, The University of Hong Kong, Prince Philip Dental Hospital, 34 Hospital Road, Sai Ying Pun, Hong Kong SAR, Peoples' Republic of China.

E-mail addresses:

| | |
|---|---|
| A Pozhitkov | pozhit@uw.edu |
| PA Noble | panoble@uw.edu |
| B Leroux | leroux@uw.edu |
| TW Randolph | trandolp@fhcrc.org |
| TF Flemmig | flemmig@hku.hk |
| T Beikler | Beikler@med.uni-duesseldorf.de |

**Contents**

Table S1. Number of sequences obtained from each condition and sample.
Table S2. Comparison of oral microbial community composition in different studies.
Figure S1. Determination of an arbitrary cut-off for displaying species on a biplot (Figure 4, right panel).

**Table S1. Number of sequences obtained from each condition and sample.**

| Condition | Gender | Label | Number of sequences | Average length bp | Average GC content |
|---|---|---|---|---|---|
| Edentulous | F | EF16 | 7,643 | 515 | 53 |
| | M | EM9 | 20,104 | 519 | 52 |
| | F | EF3 | 18,516 | 499 | 53 |
| | F | EF1 | 15,399 | 523 | 55 |
| Healthy | M | HM15 | 7,874 | 516 | 52 |
| | M | HM10 | 13,375 | 518 | 54 |
| | F | HF8 | 9,613 | 521 | 53 |
| | F | HF12 | 2,064 | 491 | 52 |
| Caries | M | CM14 | 19,131 | 518 | 53 |
| | F | CF13 | 12,870 | 521 | 52 |
| | F | CF11 | 23,904 | 500 | 52 |
| | M | CM7 | 11,532 | 519 | 54 |
| Periodontitis | F | PF6 | 7,794 | 520 | 52 |
| | F | PF5 | 13,748 | 522 | 53 |
| | M | PM4 | 12,686 | 518 | 53 |
| | M | PM2 | 13,412 | 500 | 53 |

**Table S2. Comparison of oral microbial community composition in different studies. Type, metagenome: direct extraction of DNA; 16S rRNA, extraction of DNA followed by PCR of rRNA genes; Method: cloning and Sanger, PCR amplification, cloning into plasmid vector and sequencing using the Sanger method; Subjects: H, oral health; C, caries; P, periodontitis; I, peri-implantitis; E, edentulism.**

| Study | Type | Method | Subjects | Sampling sites | PCR primers |
|---|---|---|---|---|---|
| Belda-Ferre et al. 2012 | Metagenome | 454 | 2 H, 4 C | supra-gingival plaque and cavities | None |
| Bik et al. 2010 | 16S rRNA | cloning and Sanger | 10 H | supra- sub- gingival plaque, and one saliva | 8F, 1391R |
| Diaz et al. 2012 | 16S rRNA | 454 | 5 H | saliva, buccal mucosae | V1-V2 (8F, 431R) |
| Griffen et al. 2011 | 16S rRNA | 454 | 29 H 29 P | sub-gingival samples | V1-V2 (27F, 342R), V4 |
| Gross et al. 2010 | 16S rRNA | cloning and Sanger | 18 H, 21 C | Dental plaque/lesions/carious dentin | A17, 317 |
| Gross et al. 2012 | 16S rRNA | cloning and Sanger | 36 H, 36 C | Dental plaque/lesions | A17, 317 |
| Keijser et al. 2008 | 16S rRNA | 454 | 71 H, 96 H | saliva, supra-gingival plaque | V6 (909F, 917F, 1061R) |
| Kumar et al. 2012 | 16S rRNA | 454 | 10 H, 10 P, 10 I | Subgingival, sub-mucosae | V1-V3, V7-V9 |
| Liu et al. 2012 | Metagenome and 16S rRNA | Illumina | 3H, 2P | sub-gingival plaque | 27F, 338R |
| Zaura et al. 2009 | 16S rRNA | 454 | 3 H | Supra-gingival plaque, buccal and lingual surfaces, oral mucosae, and the saliva | 785F, 1061R |
| This study | 16S rRNA | 454 | 4 H, 4P, 4C, 4 E | Supra- and subgingival plaque, oral mucosae and the saliva | 27F, 1392R |

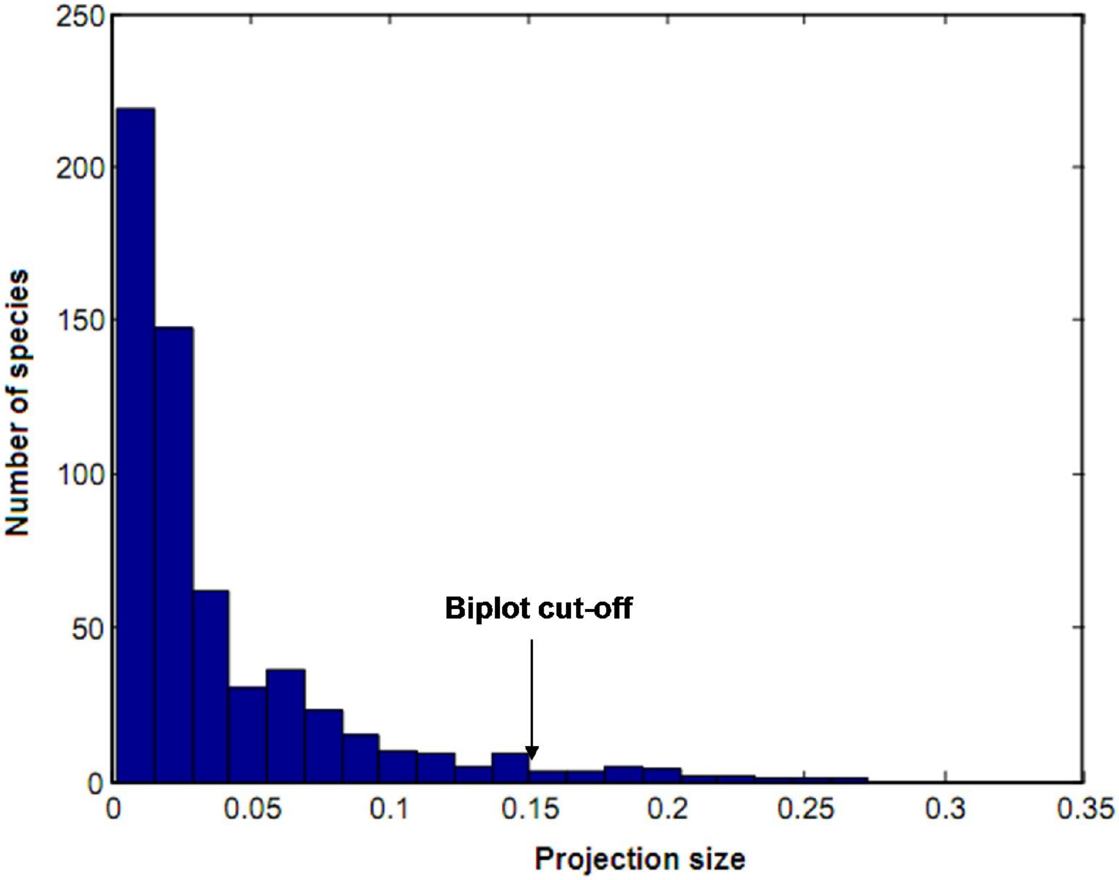


**Figure S1. Determination of an arbitrary cut-off for displaying species on a biplot (Figure 4, right panel).**
